# Kernel Density Estimation: a novel tool for visualising training intensity distribution in biathlon

**DOI:** 10.3389/fspor.2025.1546909

**Published:** 2025-06-20

**Authors:** Craig A. Staunton, Andreas Kårström, Hannes Kock, Marko S. Laaksonen, Glenn Björklund

**Affiliations:** ^1^ Department of Health Sciences, Swedish Winter Sports Research Centre, Mid Sweden University, Östersund, Sweden; ^2^ Department of Environmental and Bioscience, School of Business, Innovation and Sustainability, Halmstad University, Halmstad, Sweden; ^3^ Swedish Biathlon Federation, Östersund, Sweden; ^4^ Department of Endurance Sports, Institute for Applied Training Science, Leipzig, Germany

**Keywords:** big data, data science, density estimation, heart rate, Nordic skiing, training load

## Abstract

**Purpose:**

This study introduces two-dimensional (2D) Kernel Density Estimation (KDE) plots as a novel tool for visualising Training Intensity Distribution (TID) in biathlon. The goal was to assess how KDE plots, alongside traditional training metrics, might provide a more detailed understanding of heart rate (HR) intensity patterns, aiding in the evaluation of training quality and compliance.

**Methods:**

Fifteen elite-level youth biathletes from two national academy programmes were monitored over 5–6 weeks using HR monitors. Training sessions were measured via time-in-zone (TIZ) within a five-zone HR model with any time accumulated below the threshold for Zone 1, considered Zone 0. Sessions were dichotomised into those planned as low-intensity training (LIT) or those planned with high-intensity training (HIT). KDE analyses were conducted in MATLAB (Version R2020b) using the “*ksdensity*” function to create 2D KDE plots that visualise HR intensity accumulation across each programme, session type (e.g., Low-intensity training: LIT; High-intensity training: HIT), and individual athlete responses. Traditional histogram plots and grouped bar charts were also used for comparison.

**Results:**

For LIT sessions, athletes performed less time in Zone 1 than planned, while performed time exceeded planned time in Zone 2. For HIT sessions, performed time in Zone 5 was lower than planned. All sessions contained unplanned time in Zone 0. The 2D KDE plots provided a continuous and detailed representation of HR intensity accumulation throughout training sessions, revealing patterns and intensity fluctuations that complement traditional TIZ analyses.

**Conclusions:**

2D KDE plots might serve as a valuable complementary tool for assessing TID in biathlon, offering a more nuanced and continuous view of HR intensity. By identifying discrepancies between planned and performed training intensity, coaches can refine strategies and provide individualised feedback. Incorporating KDE plots into training monitoring could improve training alignment, helping reduce overtraining or undertraining risks and optimising athlete development.

## Introduction

Biathlon is a unique Olympic sport that combines the physically demanding discipline of cross-country skiing with the technical precision of marksmanship (1). This dual nature makes biathlon training particularly complex, as athletes must develop both exceptional endurance and skiing efficiency alongside the fine motor skills and mental focus required for accurate shooting. The sport also demands adaptability, as biathletes navigate varying terrain and employ different sub-techniques during cross-country skiing, depending on the conditions and course profile.

Training sessions in biathlon can be broadly categorised into those conducted with the biathlon rifle (WR) and those without it (No-Rifle: NR). NR training allows athletes to focus on developing physiological components, such as cardiovascular fitness, power, and efficiency in the various sub-techniques on skis or roller-skis. In contrast, WR training introduces additional layers of complexity: carrying the rifle alters skiing biomechanics (2, 3) and increases the physiological demands (4–6), while shooting practice adds a significant mental load as athletes must maintain focus and precision under physical fatigue (7, 8).

To optimise training adaptations in biathlon, it is essential to monitor both the external demands of training, such as duration, speed, or power, and the corresponding internal responses, which reflect how the body reacts to these demands (9). Internal responses are typically measured using physiological and perceptual markers like heart rate (HR), blood lactate, and ratings of perceived exertion (9, 10). In biathlon, HR monitoring is the most commonly used method for prescribing and assessing training intensity. Sessions are often structured around predefined HR-based training zones to target specific physiological adaptations. Low-intensity training (LIT), generally performed at 60%–80% of HR_max_, aims to develop aerobic capacity through prolonged efforts. In contrast, high-intensity training (HIT) involves shorter, more intense bouts designed to improve V̇O₂_max_, anaerobic capacity, and neuromuscular efficiency (11).

A widely used framework for prescribing and monitoring HR-based training is the time-in-zone (TIZ) model, which quantifies the duration spent in each training zone to describe the overall training intensity distribution (TID) (12). While TIZ offers a practical summary of the internal responses to training, this approach has several limitations. By condensing complex, continuous time-series data into simplified zone-based summaries, TIZ may result in the loss of valuable information about session dynamics (13). Moreover, aggregating separate constructs of external demand and internal response into a single summary metric can mask meaningful differences in how athletes respond to different training stimuli (14–16). As a result, two sessions with identical TIZ profiles may impose very different physiological loads (14, 17) particularly when intensity fluctuates between higher and lower zones rather than maintaining a stable, continuous effort. These fluctuations can stem from unstable internal conditions, such as a drift from low-intensity training (LIT) to high-intensity training (HIT) zones. However, they more commonly arise from external factors, such as terrain variations, which require increased effort on inclines and reduced effort on descents. These terrain-driven intensity shifts are particularly relevant in real-world settings and can lead to unintended training stimuli, despite apparent compliance with the planned duration. This underscores the need for more nuanced tools to assess session execution and overall training quality.

To address these gaps, two-dimensional (2D) Kernel Density Estimation (KDE) plots have been introduced as a novel tool for visualising the interplay between external demands and internal responses in endurance sports, such as speed skating (17). KDE is a statistical technique that estimates the probability density function of continuous data, allowing for a more nuanced representation of the coupling of external demand and internal response (18). When used alongside traditional analyses, KDE might enhance training monitoring by providing deeper insights into how training intensities are distributed over time and whether the execution aligns with intended training prescriptions.

Training quality is central to this process. Sandbakk et al. (19) describe a circular learning model for continuous improvement, in which quantitative measures of training quality assess the alignment between intended and executed training intensity. This includes how physiological markers, such as HR, deviate from planned targets. When combined with qualitative assessments of training execution, such measures provide a more holistic approach to evaluating training effectiveness.

Considering this, the purpose of this study is to explore the use of 2D KDE plots as a complementary tool for visualising TID within biathlon training programmes. By visualising TID using KDE-derived insights along with traditional TIZ metrics, this study aims to demonstrate how KDE might provide a more detailed representation of TID. In doing so, KDE analyses could offer a novel complementary approach to assessing training quality and compliance, thereby helping coaches better align performed training with planned training, optimising training prescriptions and enhancing athlete performance.

## Methods

### Participants

Fifteen elite-level youth biathletes (age 16–19 years) across two separate biathlon academy programmes (Programme 1: *n* = 6; Programme 2: *n* = 9) volunteered to participate in this study. All athletes were a part of a specialised biathlon youth academy school programme and therefore classified as Tier 3 level athletes according to the sports participant classification framework (20). The Swedish Ethical Review Authority (Dnr: 202202826-01) preapproved the research techniques and experimental protocols. All participants provided written informed consent and agreed to participate in this study. All research was conducted in accordance with the Code of Ethics of the World Medical Association (Declaration of Helsinki).

### Design

Training blocks from the two separate biathlon academy programmes were monitored, making up two out the six specialised biathlon youth academy programmes in Sweden. Training blocks for both academy programmes were monitored during similar periods of the season (February/March; competition phase). Over a 5–6-week training block, athletes wore HR monitors sampling at 1 Hz (Programme 1: H10, Polar, Finland; Programme 2: Movesense, Model: HR2, Finland) during all coach-planned training sessions, to objectively measure the athletes’ internal responses. In Programme 1, data were collected from 13 distinct training sessions, resulting in 60 individual training session observations across 6 athletes. The number of sessions contributed per athlete ranged from a minimum of 4 to a maximum of 13. In Programme 2, data were collected from 26 training sessions, yielding 63 individual training session observations from 9 athletes. Individual contributions ranged from 1 to 14 sessions per athlete.

The coaches' plan for training sessions were collected through an online training platform. This information included a plan of duration within a five-zone HR-based exercise intensity model used by the Swedish Biathlon Federation (21) (Table 1), in addition with the total planned training time. For example, a LIT session might be prescribed as “90 min in Zone 1,” while a HIT session could be planned as “20 min in Zone 4 and 60 min in Zone 2.”

**Table 1:** The five exercise intensity zones used by the Swedish biathlon federation.

Zone	Heart rate (%max)	Expected RPE	Expected blood lactate (mmol·L^−1^)
1	54–<73	10–14	<1.2
2	73–<83	14–16	1.2–<2.0
3	83–<88	16–18	2.0–<3.6
4	88–<93	18–19	3.6–<5.7
5	≥93	19–20	>5.7

RPE, rating of perceived exertion.

### Data analyses

HR data were initially trimmed to include only data captured from the beginning to the end of the training sessions. For training sessions that involved rifle shooting, the rifle “zeroing” portion of the training session was excluded. HR data from each training session were subsequently extracted and calculated as a proportion of the individual's most recently reported maximum HR (%HR_max_) and rounded to the nearest whole number. This permitted the definition of individualised training zones for each athlete, based upon their percentage of maximum HR, as outlined in Table 1. HR values falling below the lower threshold for Zone 1 were categorized as Zone 0, indicating activity below the aerobic training range.

Training sessions were dichotomised into those planned as low-intensity training (LIT) or those planned with high-intensity training (HIT). Moderate-intensity sessions were not included as a category due to the lack of sessions planned with zone 3 efforts. As such, LIT was defined as any training session planned within only HR zones 1 and 2 and had session goals of continuous, endurance-based exercise. HIT was defined as any training session that involved any planned duration within HR zone 3 or higher and were interval-based training sessions. Additionally, training sessions were categorised as those planned either no-rifle (NR) or with-rifle (WR). NR training sessions did not involve rifle carriage or shooting and was XC skiing training only. WR training sessions involved rifle carriage during XC skiing and shooting training combined with XC skiing.

### Statistical analyses

KDE analyses were performed in MATLAB (Version R2020b, The MathWorks Inc) using the “*ksdensity*” function, applying a kernel bandwidth as a smoothing parameter (22, 23). Two-dimensional (2D) KDE heatmaps were generated to visualise the distribution of time spent at each unique %HR_max_ value, expressed as a proportion of total session duration (e.g., % session time spent at 70%, 71%, 72%, etc.). Warmer colours in the heatmaps (i.e., yellow and red) indicate high density at that particular combination of % session time and %HR_max_, meaning that particular combination occurred frequently. Conversely, cooler colours (i.e., blue) represent lower density regions, meaning that particular combination occurred infrequently. White regions indicate that those combinations of % session time - %HR_max_ did not occur. These 2D KDE visualisations were used at three levels: (1) individual level – to highlight intra-athlete variability; (2) session-type level (LIT-WR, LIT-NR, HIT-WR, HIT-NR); and (3) programme-level overview. To improve interpretability and ensure that plots reflect only observed data, KDE heatmaps were capped (i.e., truncated) at the maximum observed values on both axes: %HR_max_ (*y*-axis) and time (% of session) (*x*-axis). To reduce visual complexity in the KDE heatmaps and maintain readability across figures, numerical values were intentionally omitted from the *x*-axis. Including these values would have resulted in inconsistent axis limits across plots—since maximum proportional time accumulated at any given %HR_max_ varies by session type and athlete—which could cause confusion and distract from the primary visual message. The purpose of the KDE plots is to highlight patterns of intensity distribution over time rather than to convey precise timing, which is instead provided in the accompanying histograms and TIZ plots. These complementary visualisations present the same data with numeric axes, allowing the reader to contextualise the relative timing information as needed.

Importantly, these KDE plots serve as descriptive and visual tools, intended to demonstrate a novel approach for representing training intensity distribution (TID) on a continuous scale. No inferential statistics were applied to compare training quality or compliance across groups or athletes. We explicitly caution against overinterpreting group-level KDE plots, as the unequal number of training sessions and varying data contributions across athletes can introduce bias and obscure individual patterns. This limitation is acknowledged in the discussion and further motivates the inclusion of individual-level KDEs.

To complement the 2D KDE heatmaps, one-dimensional (1D) KDE plots were also generated and displayed alongside histograms to present the frequency distribution of %HR_max_ in a more traditional format, using both discrete bins and continuous density estimates. These support the interpretation of time spent in various HR zones. Finally, grouped bar charts were included to compare planned vs. performed time-in-zone (TIZ) data.

## Results

### Training sessions

**Figure 1:**
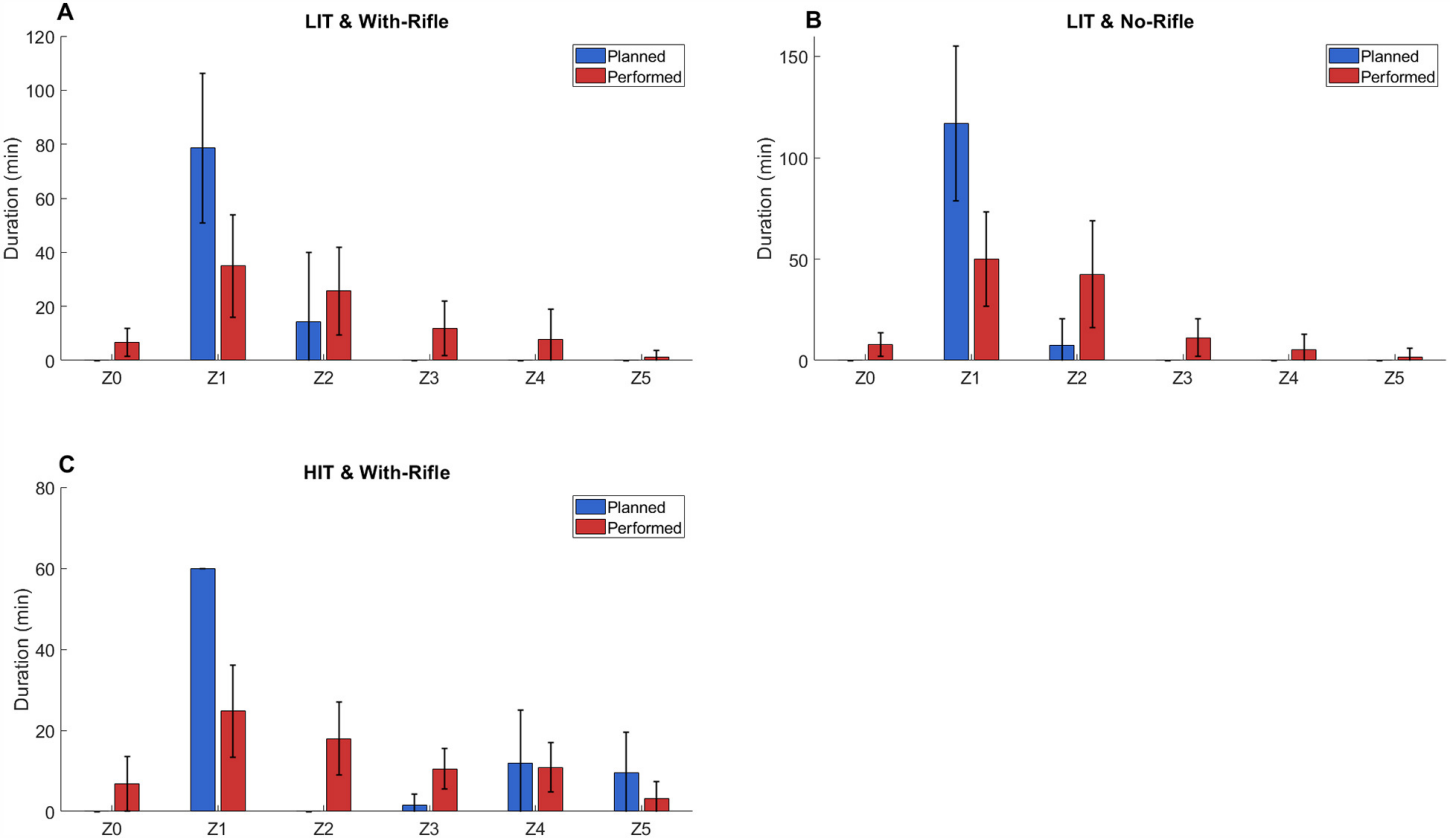
Average (± standard deviation – error bars) planned (blue) and performed (red) time in zone for all athletes for each session type for biathlon academy programme 1. LIT, low-intensity training; HIT, high-intensity training; Z0 = <54; Z1 = 54–<73; Z2 = 73–<83; Z3 = 83–<88; Z4 = 88–< 93; Z5 = ≥93 (all represent %HR_max_).

Programme 1 included 21 LIT-WR sessions (planned duration: 93 ± 61 min); 20 LIT-NR sessions (planned duration: 126 ± 38 min); 19 HIT-WR sessions (planned duration: 83 ± 5 min); and 0 HIT-NR sessions. The average planned and performed TIZ for all athletes across all training-session types for Programme 1 are shown in Figure 1. In all LIT sessions (WR and NR), athletes performed less time in Z1 than planned, while performed time exceeded planned time in Z2. In HIT sessions, performed time in Z5 was lower than planned. All sessions contained unplanned time in Z0.

**Figure 2:**
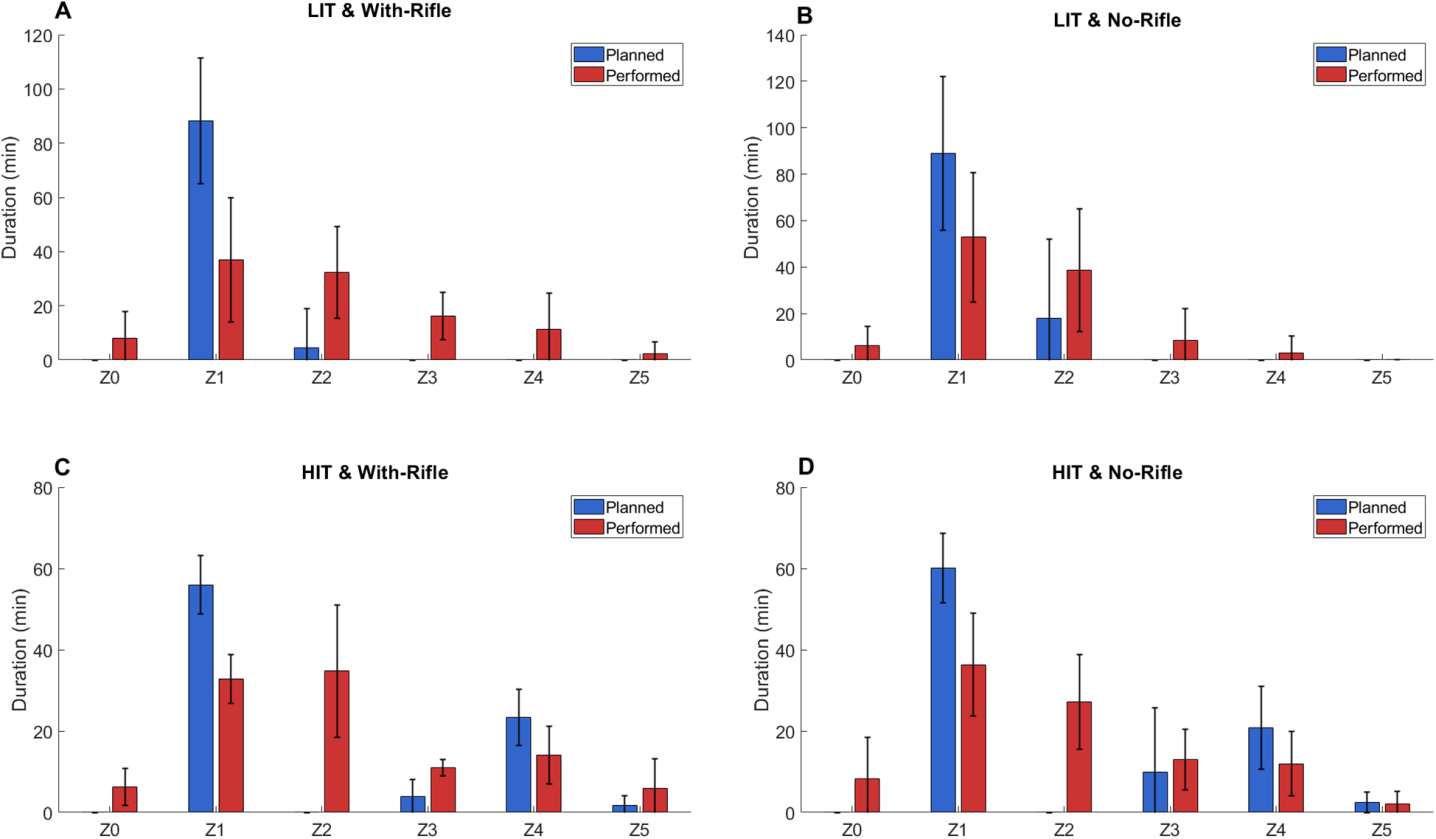
Average (± standard deviation – error bars) planned (blue) and performed (red) time in zone for all athletes for each session type for biathlon academy programme 1. LIT, low-intensity training; HIT, high-intensity training; Z0 = <54; Z1 = 54–<73; Z2 = 73–<83; Z3 = 83–<88; Z4 = 88–<93; Z5 = ≥93 (all represent %HR_max_).

Programme 2 included 11 LIT-WR sessions (planned duration: 93 ± 22 min); 34 LIT-NR session (planned duration: 107 ± 21 min); 12 HIT-NR session (planned duration: 93 ± 11 min); and 6 HIT-WR sessions (planned duration: 85 ± 8 min). The average planned and performed TIZ for all athletes across all training-session types for Programme 2 are shown in Figure 2. As with Programme 1, across all session types, athletes performed less time in Z1 than planned, while performed time exceeded planned time in Z2. In HIT sessions, performed time in Z5 was lower than planned. All sessions contained unplanned time in Z0.

### Two-dimensional kernel density estimation

#### Individual-level visualisation

**Figure 3:**
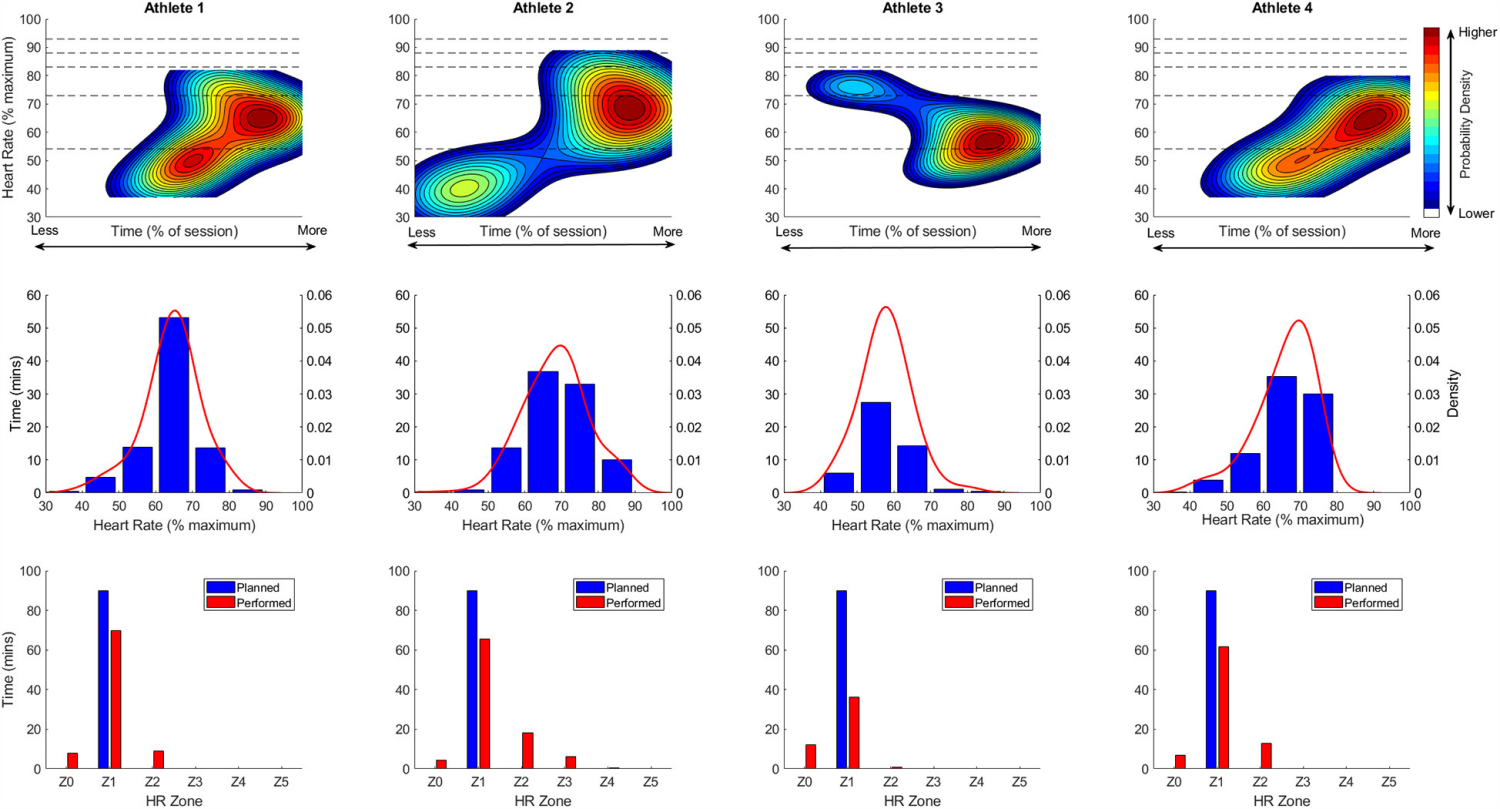
Individual session intensity plots for a single low-intensity & with-rifle session. The top row shows continuous two-dimensional (2D) Kernel Density Estimation (KDE) plots, illustrating the accumulated time spent at each unique %HR_max_ level over the course of the session. The *x*-axis represents the percentage of total session time (%), and the *y*-axis shows %HR_max_. Warmer colours (e.g., red and yellow) indicate higher probability density, i.e., where more accumulated time is concentrated at a given intensity, while cooler colours (e.g., blue and green) reflect lower probability density. Dashed horizontal lines represent the boundaries of predefined heart rate training zones. To ensure the plots reflect only observed data, the axes are capped at the maximum observed %HR_max_ (*y*-axis) and maximum accumulated time at any given %HR_max_ level (*x*-axis). The middle row presents one-dimensional (1D) KDE plots overlaid on histograms, showing the frequency distribution of accumulated time within discrete %HR_max_ bins. This provides a more traditional, discrete perspective on training intensity distribution while also including the smoothed probability density estimate. The bottom row presents grouped bar charts comparing planned vs. performed time-in-zone (TIZ).

Figure 3 presents continuous 2D KDE plots (top row), 1D KDE with corresponding histogram (middle row), and planned vs. performed TIZ (bottom row) for four individual athletes who were prescribed the same LIT-WR training session (Programme 1; planned 90 min in Z1). Despite identical training prescriptions and environmental conditions, HR distributions varied considerably among individuals. All athletes accumulated significant time in Z1 as planned; however, despite no scheduled time in Z0 or Z2, all spent some time in these zones, with Athlete 2 extending into Z3 and Z4 (Note: Athlete 3 failed to complete the full session, completing only 49 min of the prescribed 90 min).

#### Visualisation of TID between training session types

**Figure 4:**
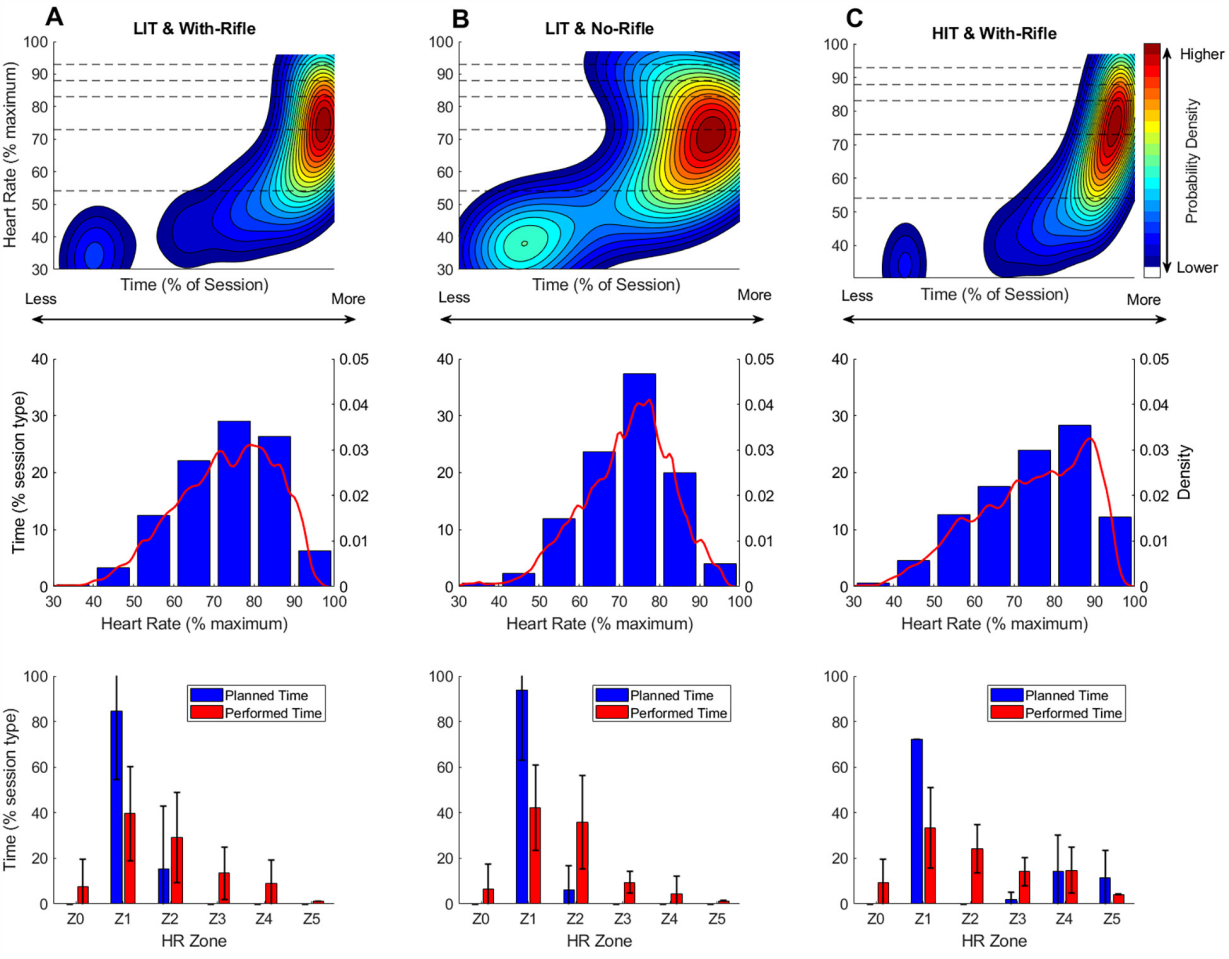
Session intensity plots for programme 1. The top row presents continuous two-dimensional (2D) Kernel Density Estimation (KDE) plots, illustrating the proportion of total session time spent at each unique %HR_max_ value across session types (LIT-NR, LIT-WR, HIT-WR; note: no HIT-NR sessions were performed in programme 1). Warmer colours (e.g., red and yellow) indicate higher probability density—i.e., more frequent occurrences of that specific %session time–%HR_max_ combination. Cooler colours (e.g., blue) indicate lower density, and white regions represent combinations of %session time and %HR_max_ that were not observed. Dashed horizontal lines mark the boundaries of predefined heart rate training zones. To ensure plots reflect only observed data, axes are capped at the maximum observed %HR_max_ (*y*-axis) and maximum accumulated session time at any given %HR_max_ value (*x*-axis). The middle row presents one-dimensional (1D) KDE plots overlaid on histograms, showing the frequency distribution of accumulated time within discrete %HR_max_ bins. This provides a more traditional, discrete perspective on training intensity distribution while also including the smoothed probability density estimate. The bottom row presents grouped bar charts comparing planned vs. performed time-in-zone (TIZ) as a percentage of total session duration. LIT-WR = 21 sessions from 6 athletes (min: 1; max 5); LIT-NR = 20 session from 6 athletes (min: 1; max: 5); HIT-WR = 19 sessions from 6 athletes (min: 1; max 5). Group-level analyses may be more influenced by some individuals who contributed more session data than others.

**Figure 5:**
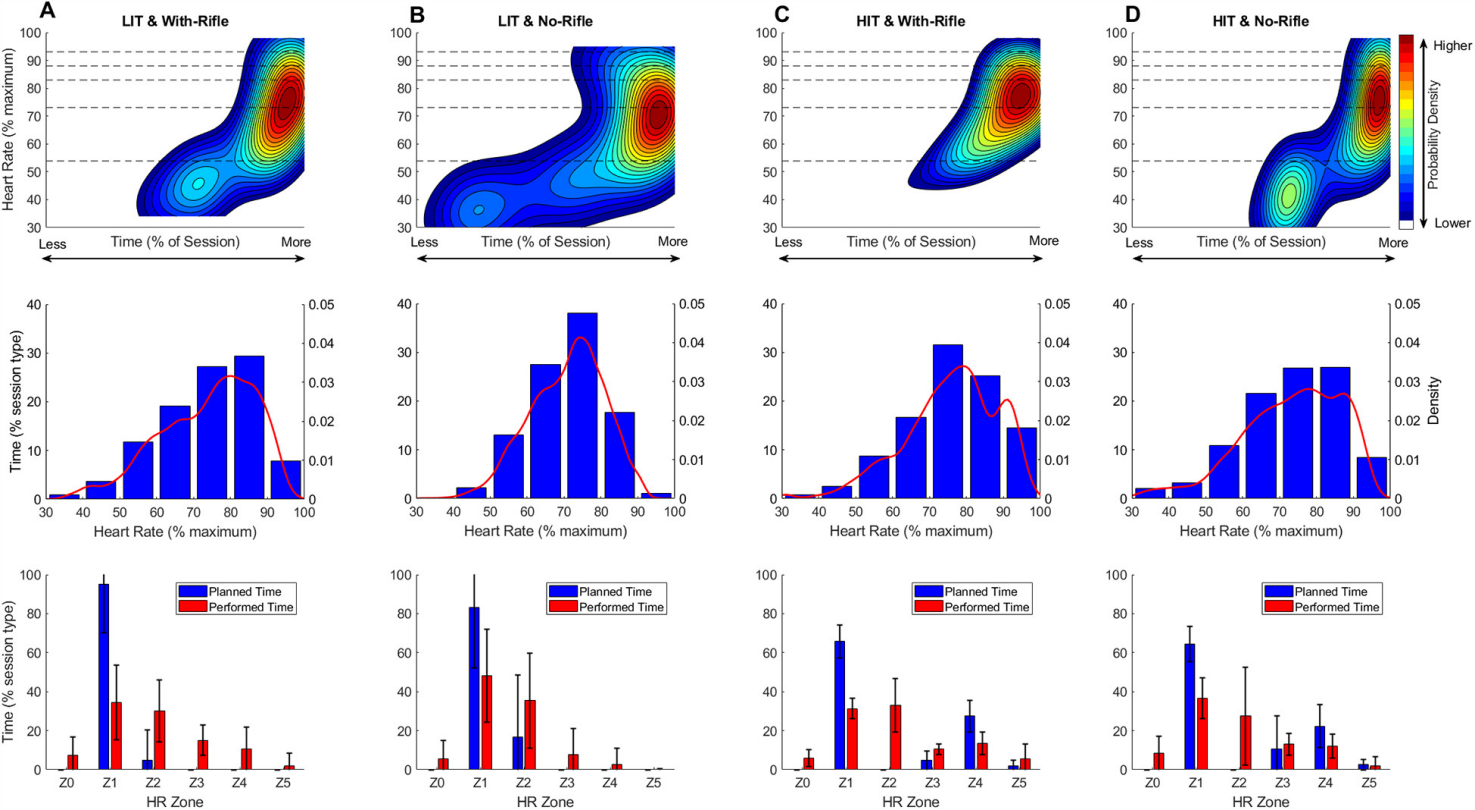
Session intensity plots for programme 2. The top row presents continuous two-dimensional (2D) Kernel Density Estimation (KDE) plots, illustrating the proportion of total session time spent at each unique %HR_max_ value across session types (LIT-WR, LIT-NR, HIT-WR, HIT-NR). Warmer colours (e.g., red and yellow) indicate higher probability density—i.e., more frequent occurrences of that specific %session time–%HR_max_ combination. Cooler colours (e.g., blue) indicate lower density, and white regions represent combinations of %session time and %HR_max_ that were not observed. Dashed horizontal lines mark the boundaries of predefined heart rate training zones. To ensure plots reflect only observed data, axes are capped at the maximum observed %HR_max_ (*y*-axis) and maximum accumulated session time at any given %HR_max_ value (*x*-axis). The middle row presents one-dimensional (1D) KDE plots overlaid on histograms, showing the frequency distribution of accumulated time within discrete %HR_max_ bins. This provides a more traditional, discrete perspective on training intensity distribution while also including the smoothed probability density estimate. The bottom row presents grouped bar charts comparing planned vs. performed time-in-zone (TIZ) as a percentage of total session duration. LIT-WR = 11 sessions from 5 athletes (min: 1; max 5); LIT-NR = 34 session from 8 athletes (min: 1; max: 9); HIT-NR = 12 sessions from 6 athletes (min: 1; max: 3); HIT-WR = 6 sessions from 3 athletes (min: 1; max 3). Group-level analyses may be more influenced by some individuals who contributed more session data than others.

Figures 4, 5 visualise the TID for each specific training session type (LIT-NR, LIT-WR, HIT-NR, HIT-WR) for both biathlon training programmes using continuous 2D KDE plots (top row), 1D KDE with corresponding histogram (middle row), and planned vs. performed TIZ (bottom row).

#### Visualisation of programme-level TID

**Figure 6:**
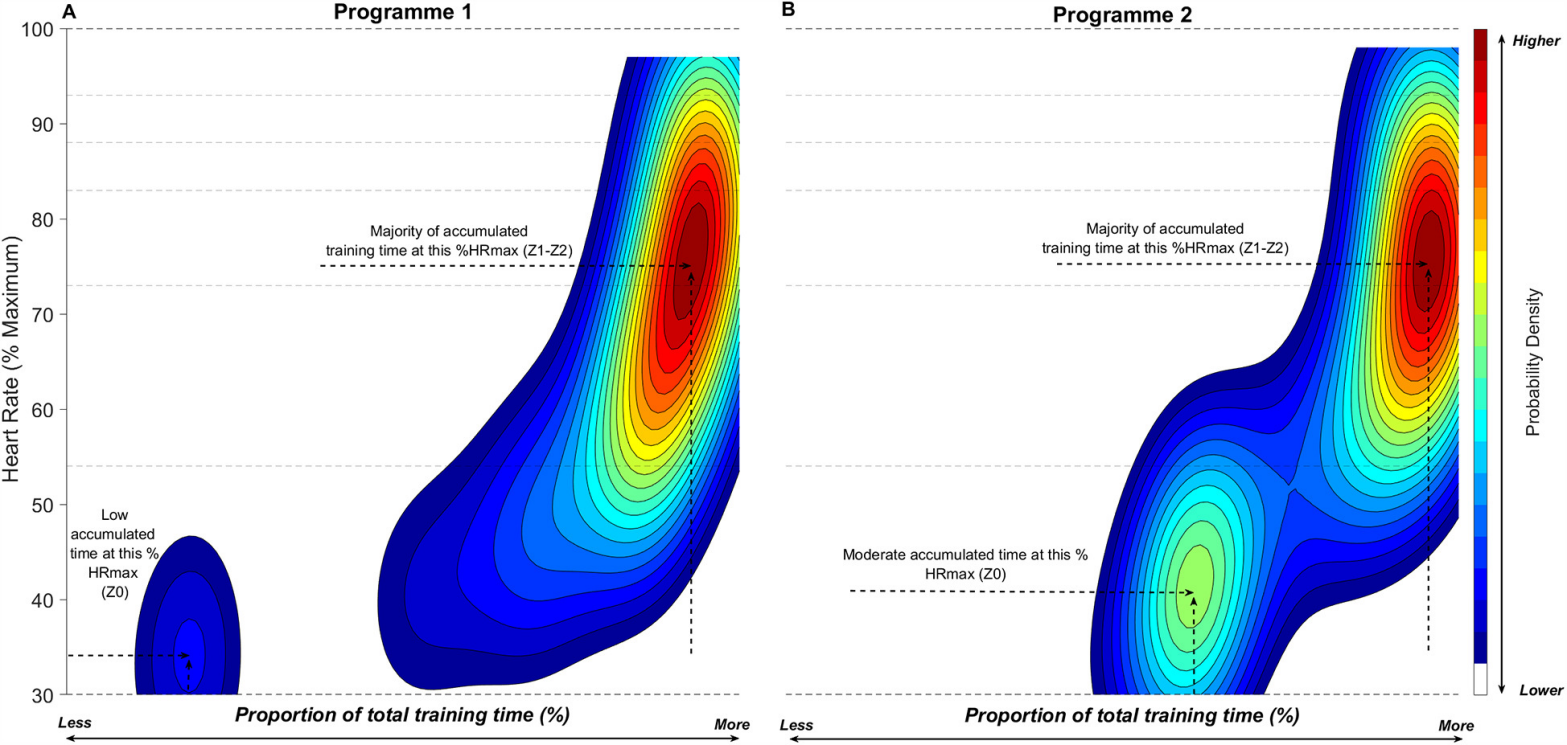
Two-dimensional kernel density estimation plots illustrating training intensity distribution for both biathlon training programmes. Continuous two-dimensional (2D) Kernel Density Estimation (KDE) plots, illustrating the proportion of total training time across all training sessions at each unique %HR_max_ value for biathlon academy programme 1 (Panel **A**) and programme 2 (Panel **B**). Warmer colours (e.g., red and yellow) indicate higher probability density—i.e., more frequent occurrences of that specific %session time–%HR_max_ combination. Cooler colours (e.g., blue) indicate lower density, and white regions represent combinations of %session time and %HR_max_ that were not observed. Dashed horizontal lines mark the boundaries of predefined heart rate training zones. To ensure plots reflect only observed data, axes are capped at the maximum observed %HR_max_ (*y*-axis) and maximum accumulated session time at any given %HR_max_ value (*x*-axis).

Figure 6 visualises the distribution of session intensity across all training sessions for biathlon programme 1 (Figure 6A) and programme 2 (Figure 6B) using 2D KDE plots, where %HR_max_ is plotted against the accumulated session duration as a proportion of total session time. Figure 6 illustrates that the 2D KDE plots reveal a more detailed visualisation of the training session intensity distributions compared with the TIZ plots (Figures 1, 2). For example, it can be observed that both programmes experience similar TID patterns, with the majority of training session duration spent at intensities corresponding with high-Z1/Z2, a tapering density at high intensities (Z4-Z5) but with a clear difference in the accumulated time at low intensity (Z0), where programme 2 has higher accumulated time in Z0.

## Discussion

This study introduced two-dimensional Kernel Density Estimation (2D KDE) plots as a novel and complementary tool for visualising training intensity distribution (TID) across three levels: the individual level – to highlight inter-individual variability in training patterns; the session-type level – to examine typical intensity distributions within different session formats; and the programme level – to provide an overarching view of intensity allocation across the full training block.

Importantly, the aim of this study was to present a novel visualisation method for examining TID patterns—not to assess training quality or compliance in this specific population. However, by integrating KDE with traditional training metrics, this approach might offer a more detailed assessment of training quality, revealing whether sessions were performed as intended. Coaches and athletes might be able to use these insights to evaluate alignment between planned and executed training, identify deviations that may impact adaptation, and refine training prescriptions to optimise performance outcomes.

### Individual response level

The combination of KDE plots, histograms, and TIZ analyses offers a comprehensive visualisation of how training intensity was planned and executed. For example, Figure 3 depicts the TID for four different athletes who were prescribed the same LIT-WR training session (Programme 1: planned time 90 min Z1).

Despite identical training prescriptions and environmental conditions, HR distributions varied considerably among individuals. All athletes accumulated significant time in Z1 as planned; however, despite no scheduled time in Z0 or Z2, all spent some time in these zones. Athlete 1 exhibited a high-density heat spot in the middle of Z1 (as per planned training) but also along the Z0–Z1 boundary, suggesting frequent transitions between these zones, and the high-density heat spot extending in Z2. This might indicate poor pacing during undulating terrain where the athlete struggled to stay within Z1 during uphill and downhill sections. For example, high-level coaches have discussed that it may be necessary to move into Z2 HR to maintain good technique on uphill sections (24). Additionally, shooting practice likely contributed to the unplanned higher-density heat spot visible in Z0. In contrast, Athlete 2 displayed a bimodal distribution with a primary high-density heat spot on the upper border of Z1 but a secondary heat spot in the lower left of the plot, suggesting an initial phase at very low intensities and/or un-planned recovery periods. This athlete accumulated the least time in Z0 but spent the longest duration in Z2 and Z3. Athlete 3 accumulated most of their training time in the lower portion of Zone 1. The main heat spot extended into Zone 0, indicating a significant amount of time spent at low intensity. Despite completing only 49 of the prescribed 90 min, Athlete 3 accumulated over 10 min in Zone 0, the longest duration observed in this zone. A secondary, less prominent heat spot appeared in Zone 2, suggesting a brief period of higher-intensity effort. Athlete 4 demonstrated a more dispersed distribution of HR data, ranging from Z0 to Z2 but centred in Z1, reflecting greater variability while still aligning with the intended training intensity.

Taken together, these findings highlight notable inter-individual differences in HR responses, even when prescribed the same structured training session and the same external environmental conditions, emphasising the potential influence of physiological and behavioural factors on training execution.

Beyond a single session, the individual-level HR responses can also be applied on a larger scale to help evaluate training execution at the session-type and programme levels. By aggregating individual responses across multiple sessions, it becomes possible to compare how different athletes execute the same session type or even how they perform throughout an entire training programme. This allows for a more detailed comparison of training execution, identifying whether certain athletes consistently struggle to maintain prescribed intensities or whether they frequently exceed or underperform in specific HR zones.

For example, at the session-type level, coaches or sports scientists could use these KDE plots to assess whether athletes tend to spend too much time in lower (Z0, Z1) or higher (Z2, Z3) zones, deviating from the intended intensity. This could highlight issues such as pacing difficulties, insufficient recovery, or even an athlete's unplanned fatigue accumulation. Similarly, across a training programme, the cumulative data could reveal trends over time, such as consistent deviations or improvements in maintaining the prescribed intensity, providing valuable insights into the athlete's adaptation to training.

### Session-type level

Across both biathlon academy programmes, training prescriptions for each session type were generally similar, and the execution followed comparable patterns. For instance, LIT-WR sessions (Figures 4A, 5A) were primarily prescribed in Z1, with a smaller proportion in Z2, and no planned training in Z0 or Z3–Z5. The KDE plots for these sessions illustrate a bimodal intensity distribution, with the majority of training time accumulating at 70%–80% HR_max_ (borderline Z1/Z2), and a secondary, lower-intensity peak in Z0. This bimodal pattern is confirmed by the histograms, which highlight that the majority of the training time is spent in the 70%–80% HRmax bin. However, the histogram also suggests some distribution into the 80%–90% HRmax bin, though much of it is likely within the 80%–83% HRmax range—still within Z2, not Z3. While no training time was explicitly planned for Z0, its presence might be reasonably expected due to the inclusion of shooting practice in WR sessions, pointing to the possibility that this factor is not always accounted for in training prescriptions.

In contrast, LIT-NR sessions (Figures 4B, 5B), designed for continuous low-intensity endurance, also display a bimodal distribution. Ideally, a well-executed LIT session would show a single high-density heat spot in the low-intensity zones (Z1–Z2), with minimal or no time spent in higher intensity zones (Z3–Z5). The presence of deviations from this pattern, such as time spent in Z3–Z4 or excessive time in Z0, could indicate issues such as difficulty maintaining intensity due to terrain, pacing difficulties, or environmental factors that influence intensity control. The KDE plots here offer a visual representation of these potential deviations, which might otherwise be overlooked in summary statistics.

For HIT sessions, KDE plots are expected to display distinct peaks corresponding to alternating high-intensity work intervals and low-intensity recovery periods. According to the planned training, high-density heat spots would be expected in Z3–Z5 during work intervals and in Z1 during recovery, with minimal time spent in Z2. Deviations from this expected pattern, such as a lack of clear separation between work and recovery intervals or an accumulation of training time in Z2 instead of Z4/Z5, can be visualised in the KDE plots.

The “regression toward the mean” pattern is a well-documented training error among endurance athletes, where high-intensity sessions are performed at a lower intensity than intended, while low-intensity sessions drift too high (25). Similar discrepancies between planned and executed training intensity have been observed across various sports, including endurance sports such as cycling (26) and swimming (27) and team sports, like basketball (28) and tennis (29).

A key factor influencing recovery is rifle carriage (WR). Figures 4C, 5C depict the TID for HIT-WR sessions. Here it is apparent that WR limits recovery, as indicated by the lack of a bimodal pattern. Instead of bimodal heat spots between Z1 and Z4 (as per the training plan), the KDE density extends downward through Z3 and Z2, which might indicate that HR remains elevated during recovery periods but also represents natural physiological HR recovery from intervals. Furthermore, in both LIT and HIT sessions, WR conditions shift the HR distribution upward compared to NR conditions, extending previous research findings that indicate rifle carriage not only elevates HR but also reduces recovery periods (2, 4, 5). This effect likely increases overall session intensity and physiological strain, influencing how training sessions are executed relative to their intended TID.

In summary, the KDE plots might offer a novel and complementary tool for visualising TID at the session-type level. By providing a detailed, real-time snapshot of how training is executed relative to planned intensities, these plots allow coaches and athletes to identify patterns and deviations that may not be immediately apparent through traditional metrics. This visualisation tool can help enhance the understanding of TID, offering a more dynamic and individualized approach to monitoring and refining training strategies.

### Programme level

At the programme level, the 2D KDE plots (Figure 6) offer valuable insights into the overall distribution of time spent across various %HR_max_ intervals for all training sessions within each biathlon academy programme. For example, Programme 2 displays a notable accumulation of time in Z0, which is particularly prominent in the KDE plots. This finding is intriguing, especially given the lower proportion of HIT and WR sessions in Programme 2, as these session types typically involve some time in Z0, even if not explicitly planned. A possible contributing factor is the elevation difference between the shooting ranges in the two programmes: in Programme 1, the shooting range was located at a lower elevation, while in Programme 2, it was situated at a higher elevation. This elevation difference may influence recovery periods, impacting TID and providing a further dimension for coaches to consider when prescribing and monitoring training.

From a broader perspective, KDE plots provide a complementary tool for assessing the alignment of training execution with the intended TID models across programmes. In a “polarized” training programme, the KDE plot should exhibit a distinct bimodal distribution, reflecting the characteristic division between LIT and HIT, with minimal time spent in moderate-intensity zones. In contrast, Figure 6 visualises the typical TID of a “pyramidal” training programme, where the KDE plot presents a gradual distribution of time across intensities. Both programmes display high-density heat spots in the lower-intensity zones (Z1–Z2), moderate density in the mid-intensity zone (Z3), and a tapering density in the high-intensity zones (Z4–Z5).

These visualisations offer a comprehensive tool for coaches, enabling them to assess not only how training sessions are executed but also how they compare across different programmes. By using KDE plots to monitor TID, coaches can identify areas for improvement in training execution, fine-tune prescriptions, and ultimately ensure that training is aligned with the planned intensity model. This approach can help reduce discrepancies between the prescribed and performed TID, enhancing the overall quality of the training programme and contributing to more effective training adaptations.

## Practical applications – implications for training monitoring

This study illustrates the use of 1D and 2D KDE plots as a complementary tool for visualising TID in biathlon. The 1D KDE plot shows the estimated density of a single variable (i.e., %HR_max_), while the 2D KDE plot estimates the joint density of two simultaneous variables (i.e., the combination of %HR_max_ and %session time). When used alongside traditional metrics like TIZ analyses, KDE plots offer a more detailed, continuous view of HR magnitude and duration for individuals, training session types and training programmes.

Unlike TIZ or histogram-based methods, which divide HR data into fixed zones, KDE plots show intensity as a smooth, continuous distribution. This enables a more nuanced understanding of how training intensity fluctuates throughout a session and might reveal patterns that could otherwise be missed.

KDE plots could be particularly useful as a complementary tool for coaches aiming to evaluate and fine-tune training execution. They can help to:

• Support individualised feedback: Compare how different athletes respond to training and adjust future prescriptions accordingly. Individual-level analyses should be completed on single training sessions and also on combined data from all sessions of a given type. This could be extended across the individual's total training across all session types to provide the best feedback regarding training alignment.
• Identify mismatches between planned and performed sessions: Detect deviations from intended TID in specific session types (e.g., LIT vs. HIT, WR vs. NR).
• Assess programme-level TID patterns: For example, verify whether a program follows a polarized or pyramidal model by visualising time spent in low, moderate, and high intensity zones.

By integrating KDE plots into the circular learning process described by Sandbakk et al. (19), coaches could make data-informed adjustments to improve training alignment and quality over time. Thereby, KDE plots offer a novel approach which might help reduce the risk of unintentional over- or under-training., ultimately supporting better decision-making and enhancing athlete development.

## Limitations and perspectives

The intensity distribution patterns displayed in this study are based purely upon internal responses (i.e., HR). It is important to consider that such responses are subject to delayed responsiveness to changes in intensity due to physiological lag (6). Conversely, analysis of the TID patterns based upon external demand might produce a different TID pattern. In fact, previous research has shown that TID can vary when measured as external demand (power output) or internal response (HR) (30). Despite these known limitations, internal responses (HR) were measured in this study because this approach remains the most common approach to training intensity quantification in biathlon. Although estimates of power output are possible using wearable sensor technology, these approaches are not commonly adopted in practice.

Additionally, an important consideration is that the group-level analyses presented in the present study may be more influenced by some individuals who contributed more session data than others. No weighting or normalisation procedures were applied to balance contributions from each athlete. Individual-level contributions should be more carefully controlled in future applications of this method, particularly in studies aiming to compare training strategies or outcomes across groups. However, it was not the aim of this research to evaluate training compliance or quality but rather to present a novel method to visualise TID, which, in turn, might help to evaluate these factors.

Finally, the small sample size used in this study limits the generalisability. Future research with a larger and more diverse group of athletes can help to further validate these insights and enhance their applicability to broader populations.

## Conclusion

This study demonstrates that 1D and 2D KDE plots could offer a complementary approach to traditional methods of visualising training demands. While traditional metrics like TIZ or histograms focus on summarising data into discrete bins, KDE plots provide a continuous representation of HR variations across training sessions. This added layer of detail might allow coaches to gain a deeper understanding of TID, enabling data-informed adjustments to improve training alignment and quality. By incorporating KDE plots alongside existing tools, coaches could enhance their ability to match performed training with the planned intensity, helping to reduce the risks associated with overtraining or undertraining. Ultimately, KDE plots have potential to serve as a valuable complement to traditional training metrics, providing a more comprehensive view of TID.

## Data Availability

The raw data supporting the conclusions of this article will be made available by the authors, without undue reservation.
